# A huge hepatic angiomyolipoma with growth during 5 years of follow-up

**DOI:** 10.1093/jscr/rjaa353

**Published:** 2020-09-25

**Authors:** Michio Machida, Hiroyuki Sugo, Ikuo Watanobe

**Affiliations:** Department of General Surgery, Juntendo University Nerima Hospital, Nerima-ku, Tokyo, Japan; Department of General Surgery, Juntendo University Nerima Hospital, Nerima-ku, Tokyo, Japan; Department of General Surgery, Juntendo University Nerima Hospital, Nerima-ku, Tokyo, Japan

## Abstract

A 45-year-old woman was referred to our hospital with a huge liver tumor that had been diagnosed as a hepatic angiomyolipoma (HAML) 5 years previously. At the time of referral, it had enlarged from 12 to 20 cm within the previous 5 years and become symptomatic. Enhanced computed tomography revealed a very large, well-defined, low-density mass occupying the entire right lobe of the liver. The patient underwent right hemi-hepatectomy. The resected specimen weighed 1620 g and measured 20 × 14 × 8 cm. The pathological diagnosis was confirmed as benign HAML. The estimated growth rate of this tumor was 44% per year with a doubling time of 826 days. Although the majority of HAMLs are stable lesions, resection should perhaps be considered when the tumor is known to be growing and exceeds 6 cm in diameter, even if it has been diagnosed as benign.

## INTRODUCTION

Hepatic angiomyolipoma (HAML) is a rare, benign hepatic tumor characterized by a content of lipomatous, myomatous and angiomatous tissues [1]. Unlike kidney AMLs, the vast majority of HAMLs is stable lesions, and should be followed up by observation in principle. In a few cases, however, changes in size and internal composition have been observed. Here we describe a patient with a huge HAML that had enlarged over a 5-year period since initial diagnosis.

## CASE REPORT

**Figure 1:**
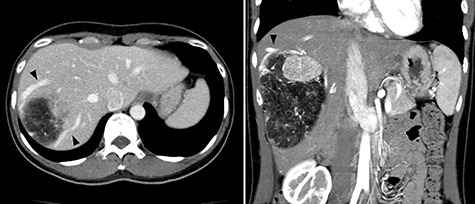
Abdominal computed tomography (CT) 5 years before referral to our hospital; enhanced CT revealed mixed density tumor measuring 12 × 10 cm in the right lobe of the liver; a peritumoral early drainage vein around the tumor (arrow heads) was visualized.

**Figure 2:**
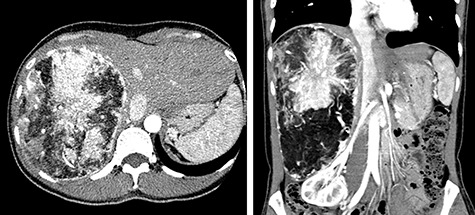
Abdominal CT on admission; the tumor had enlarged, occupying the entire right lobe of the liver, and measuring 200 × 140 × 120 mm.

**Figure 3:**
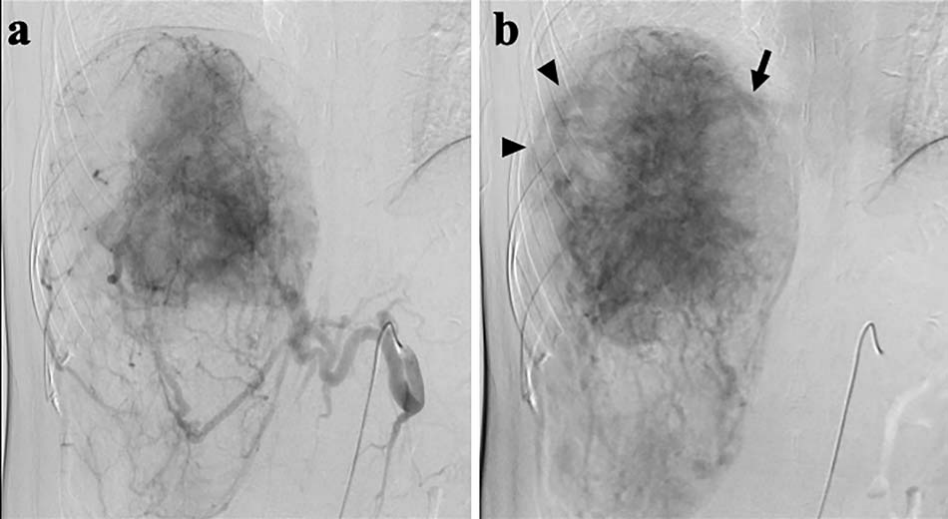
Abdominal angiography; (**a**) hepatic angiography shows a hypervascular tumor with a fine network of vessels in the arterial phase; (**b**) dilated and tortuous vessels (arrow heads) were noted inside the tumor, then a drainage vein to the right hepatic vein (arrow) was visualized in the early phase.

A 45-year-old woman was referred to our hospital with a huge liver tumor, which had been diagnosed as HAML 5 years previously when it was 12 cm in diameter (Fig. 1). Periodic follow-up with ultrasound and magnetic resonance imaging after the initial diagnosis had shown a progressive increase in the size of the tumor, and by the time of referral it measured 20 × 14 cm and had become symptomatic. On admission, enhanced computed tomography revealed a very large, well-defined, mixed-density mass occupying the entire right lobe of the liver (Fig. 2). Angiography showed dilated and tortuous vessel inside the tumor, and a drainage vein from the tumor to the right hepatic vein was visualized in the early phase (Fig. 3).

**Figure 4:**
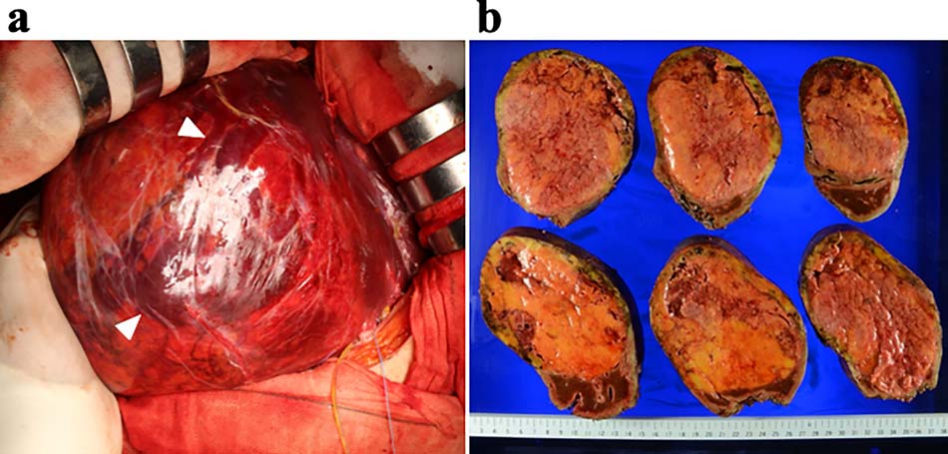
Intraoperative findings (**a**) and the resected specimen (**b**); (a) a dilated abdominal vein was observed on the surface of the liver (arrows); (b) the cut surface of the resected specimen revealed a heterogenous appearance with areas of hemorrhage (red) and fatty components (yellow) with a capsule.

**Figure 5:**
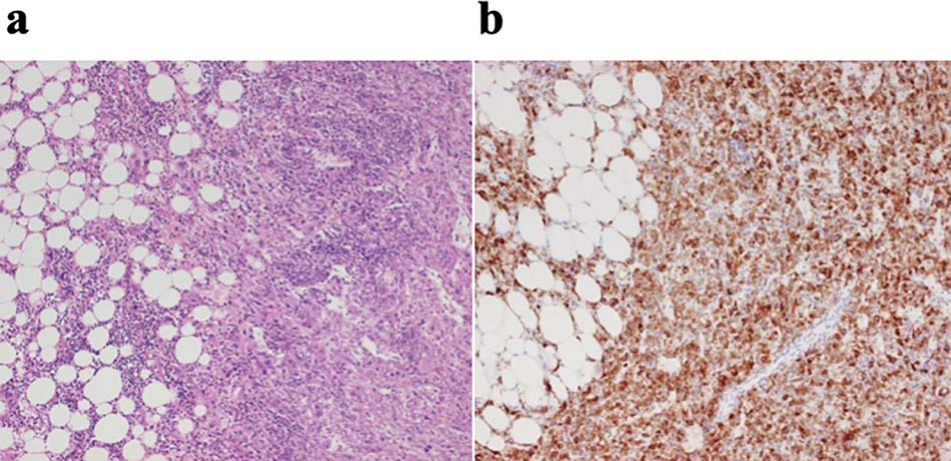
Histopathological findings; (**a**) the tumor was composed of mature lipocytes with angiomatous and small lymphocytic components, but showed no mitotic figures; (**b**) immnohistochemically, the tumor was positive for HMB45.

The patient underwent right hemi-hepatectomy. Intraoperatively, a relatively soft dark red giant tumor about 20 cm in diameter was found to occupy the whole right lobe of the liver, and a dilated abnormal vein was observed on the liver surface (Fig. 4a); the resected specimen weighed 1620 g and measured 20 × 14 × 8 cm. The cut surface of the resected specimen revealed a heterogenous appearance with areas of hemorrhage and fatty components with a capsule (Fig. 4b). The pathological diagnosis was confirmed as HAML with areas of enriched vessels, extramedullary hematopoiesis, fatty tissue and mostly proliferative epithelioid cells immunoreactive for homatropine methylbromide 45 (HMB45) (Fig. 5). The postoperative course was uneventful, and the patient was discharged on postoperative Day 10.

## DISCUSSION

Radiological diagnosis of HAML is occasionally difficult since the proportion of adipose tissue in the tumor varies greatly, ranging from 5 to 90% [1]. The typical radiological criteria for HAMLs are: (i) high vascularization of in a solid tumor and (ii) presence of macroscopic fatty components [2]. An early drainage vein around the tumor may also be very helpful for diagnosis [3–4]. In a study of 47 patients with HAML, Nonomura *et al*. [5] demonstrated that abnormally large, dilated blood vessels were present around the tumors at the tumor-background liver interface in 40% of the HAMLs they examined. This feature was not found in tumors < 25 mm in diameter, but was present in 60% of HAMLs with a diameter of ≥25 mm. In the present case, imaging modalities showed these typical findings, enabling the diagnosis of HAML.

**Figure 6:**
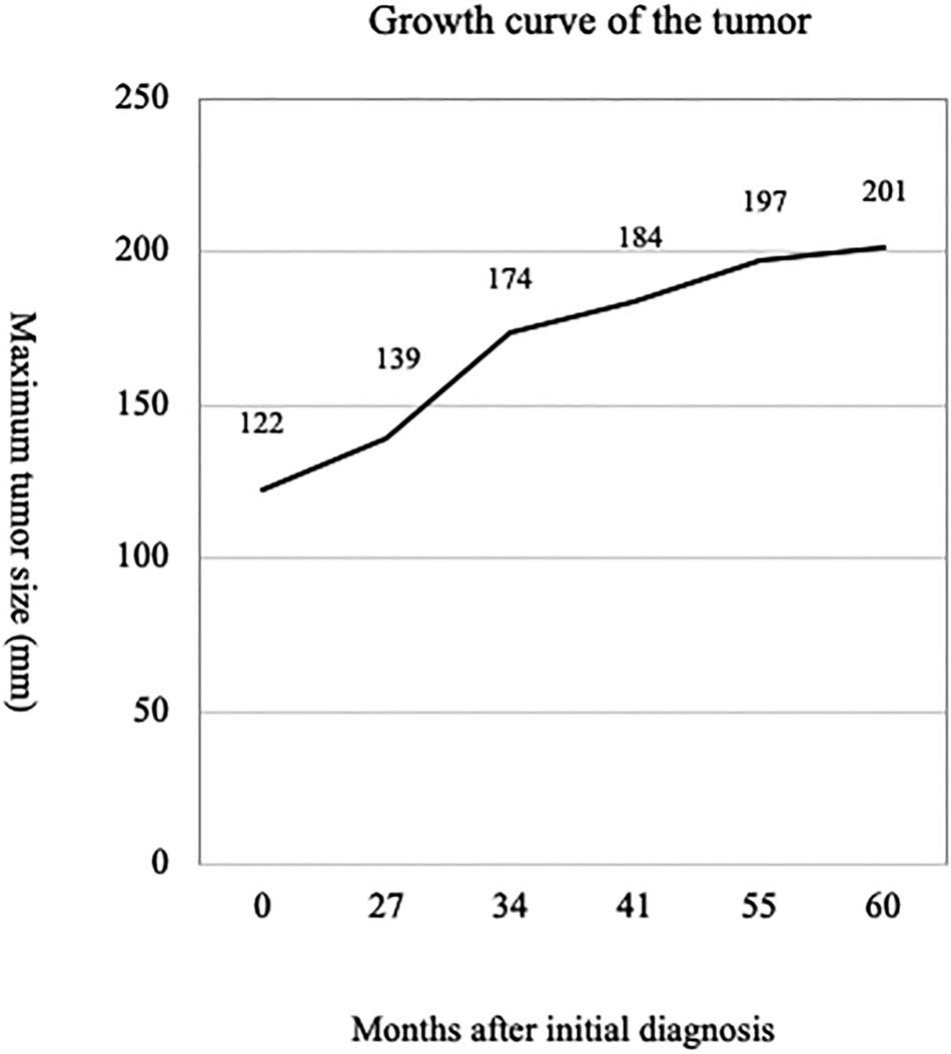
Growth curve of the tumor.

In the past, HAML was considered a benign tumor that could be managed conservatively. On the other hand, a small number of reported cases exhibit malignant characteristics, such as invasive growth pattern, vascular invasion and local recurrence after curative resection, as well as distant metastasis. The growth rate and the presence of atypical cells are more critical for estimating the malignant potential of this type of tumor rather than size alone. In our patient, the tumor had enlarged from 12 to 20 cm within the previous 5 years and become symptomatic. The tumor growth was progressive and quite constant, at least based on images obtained at the previous hospital (Fig. 6), but neither metastasis nor invasive growth were evident so far. The estimated growth rate of the tumor was 44% per year with a doubling time of 826 days.

Including the present case, only 19 cases of HAML with growth have been reported in the Japanese literature [6, 7]. A review of these 19 cases revealed that the average size of the tumor was 66 mm, ranging from 16 to 200 mm, and their mean doubling time was 460 days, ranging from 61 to 2094 days. On the other hand, Mizuguchi *et al*. [8] reported a malignant HAML with a tumor doubling time of 78.9 days. The median doubling time of hepatocellular carcinoma showing rapid growth was reported to be 56 days [9]. Although the present HAML had enlarged from 12 to 20 cm in 5 years with a doubling time of 826 days, it was considered to have grown rather more slowly than usual.

On the other hand, another report has suggested that a surgical strategy should be adopted for (i) all patients with symptoms, (ii) patients with tumors more than 6 cm in diameter, (iii) patients with tumors showing extra-hepatic growth or risk of rupture, (iv) patients with growing tumors and (v) patients for whom imaging modalities and/or biopsy examination have not produced a definitive diagnosis [10]. Obviously, early surgical intervention may have been considered appropriate for the present case, even though the tumor was benign and its growth rate not so high. Currently, laparoscopic resection is the first-choice treatment for HAML, as it is less invasive with fewer postoperative complications. However, these advantages are lost once the tumor has grown beyond the size manageable using a laparoscopic approach. To our knowledge, the present tumor 20 cm in diameter and weighing 1620 g is the largest HAML to have been reported so far, and consequently major hepatectomy via laparotomy was required.

## CONCLUSION

Although the majority of HAMLs are stable lesions, resection should perhaps be considered when the tumor is known to be growing and exceeds 6 cm in diameter, even if it has been diagnosed as benign.
